# A comparative study of altered hemodynamics in iliac vein compression syndrome

**DOI:** 10.3389/fbioe.2024.1302063

**Published:** 2024-01-18

**Authors:** Ismael Z. Assi, Sabrina R. Lynch, Brian D. Ricker, Siddhant V. Ranjane, David M. Williams, Thomas W. Wakefield, Andrea T. Obi, C. Alberto Figueroa

**Affiliations:** ^1^ Department of Biomedical Engineering, University of Michigan, Ann Arbor, MI, United States; ^2^ School of Arts and Sciences, Johns Hopkins University, Baltimore, MD, United States; ^3^ Division of Vascular and Interventional Radiology, Department of Radiology, University of Michigan, Ann Arbor, MI, United States; ^4^ Section of Vascular Surgery, Department of Surgery, University of Michigan, Ann Arbor, MI, United States

**Keywords:** iliac vein compression syndrome, May-Thurner syndrome, Cockett syndrome, computational fluid dynamics, thrombosis, shear rate, patient-specific modeling

## Abstract

**Introduction:** Iliac vein compression syndrome (IVCS) is present in over 20% of the population and is associated with left leg pain, swelling, and thrombosis. IVCS symptoms are thought to be induced by altered pelvic hemodynamics, however, there currently exists a knowledge gap on the hemodynamic differences between IVCS and healthy patients. To elucidate those differences, we carried out a patient-specific, computational modeling comparative study.

**Methods:** Computed tomography and ultrasound velocity and area data were used to build and validate computational models for a cohort of IVCS (N = 4, Subject group) and control (N = 4, Control group) patients. Flow, cross-sectional area, and shear rate were compared between the right common iliac vein (RCIV) and left common iliac vein (LCIV) for each group and between the Subject and Control groups for the same vessel.

**Results:** For the IVCS patients, LCIV mean shear rate was higher than RCIV mean shear rate (550 ± 103 s^−1^ vs. 113 ± 48 s^−1^, *p* = 0.0009). Furthermore, LCIV mean shear rate was higher in the Subject group than in the Control group (550 ± 103 s^−1^ vs. 75 ± 37 s^−1^, *p* = 0.0001). Lastly, the LCIV/RCIV shear rate ratio was 4.6 times greater in the Subject group than in the Control group (6.56 ± 0.9 vs. 1.43 ± 0.6, *p* = 0.00008).

**Discussion:** Our analyses revealed that IVCS patients have elevated shear rates which may explain a higher thrombosis risk and suggest that their thrombus initiation process may share aspects of arterial thrombosis. We have identified hemodynamic metrics that revealed profound differences between IVCS patients and Controls, and between RCIV and LCIV in the IVCS patients. Based on these metrics, we propose that non-invasive measurement of shear rate may aid with stratification of patients with moderate compression in which treatment is highly variable. More investigation is needed to assess the prognostic value of shear rate and shear rate ratio as clinical metrics and to understand the mechanisms of thrombus formation in IVCS patients.

## 1 Introduction

Iliac vein compression syndrome (IVCS), also known as May-Thurner syndrome or Cockett syndrome, is an anatomical compression of the left common iliac vein (LCIV) by the right common iliac artery against the lumbar spine (May and Thurner, 1957) resulting in a spectrum ranging from no symptoms, to left leg swelling and pain, to venous thrombosis. Iliac vein compression has been shown to occur in over 20% of the population in asymptomatic individuals (May and Thurner, 1957; Kibbe et al., 2004).

To elucidate the mechanism by which IVCS predisposes patients to venous thrombosis, several studies have investigated the effect of IVCS on iliac vein hemodynamics. Ultrasound studies have demonstrated that anatomical compression of the LCIV increases post-stenotic blood velocities by as much as 3-8x (Labropoulos et al., 2007; Oğuzkurt et al., 2007; Cheng et al., 2017; Engelhorn et al., 2021). Computational fluid dynamics studies have further demonstrated that IVCS leads to increased blood velocities, wall shear stresses, and pressure gradients in the stenosed vessel (Wang et al., 2022; Assi et al., 2023; Li et al., 2023).

Despite these studies establishing several key hemodynamic features of IVCS, there currently exists a lack of information on the hemodynamic differences between IVCS and healthy patients. Furthermore, there is a need to develop a quantitative and repeatable metric that can characterize the clinical significance of a venous stenosis. This metric would be especially relevant for patients with moderate, yet symptomatic compression for which there is considerable variability in clinical management (Hng et al., 2021). Therefore, in this work, we carried out a patient-specific, computational modeling comparative study to: A) identify hemodynamic differences between IVCS and healthy patients, B) develop a clinical metric to quantitatively characterize iliac vein hemodynamics, and C) hypothesize how hemodynamic differences between stenosed and healthy vessels may predispose IVCS patients to venous thrombosis.

## 2 Materials and methods

The methods for clinical data acquisition and computational modeling in this study were tailored from our recently developed iliac vein computational modeling protocol (Assi et al., 2023).

### 2.1 Clinical data

This single-center, non-randomized, case-control study was approved by the University of Michigan Institutional Review Board (HUM00212189). Informed consent was obtained from all study participants. Clinical data was acquired from a cohort of four patients with IVCS and DVT (deep vein thrombosis) and/or lower extremity venous symptoms (**Subject Group**) and four patients with arterial disease without lower extremity venous symptoms and no IVCS or DVT (**Control Group**). Table 1 contains patient demographics for the Subject and Control groups.

**TABLE 1:** Patient demographics for the Subject and Control groups. There was a statistically significant difference in age, and no statistically significant differences in sex, race, height, weight, BMI, or respiratory lengths between the two groups.

Patient demographics	Subjects				Average	Std	Controls				Average	Std	*p*-value	
	#1	#2	#3	#4			#1	#2	#3	#4				
Age (years)	25	41	52	47	41.3	11.7	85	62	62	61	67.5	11.7	0.019	*
Sex	F	F	M	F	N/A	N/A	M	F	F	F	N/A	N/A	1.000	ns
Race	White	White	White	White	N/A	N/A	White	White	White	White	N/A	N/A	1.000	ns
Height (m)	1.59	1.73	1.83	1.65	1.7	0.1	1.78	1.65	1.65	1.63	1.7	0.1	0.731	ns
Weight (kg)	56.3	104.2	73.0	81.6	78.8	19.9	93.2	112.0	80.4	111.4	99.3	15.3	0.154	ns
BMI	22.3	34.9	21.8	29.9	27.2	6.3	29.4	41.1	29.5	42.1	35.5	7.0	0.129	ns
Respiratory Length (s)	3	3	3.75	3.2	3.2	0.4	3	3.75	3.5	3	3.3	0.4	0.781	ns

**FIGURE 1:**
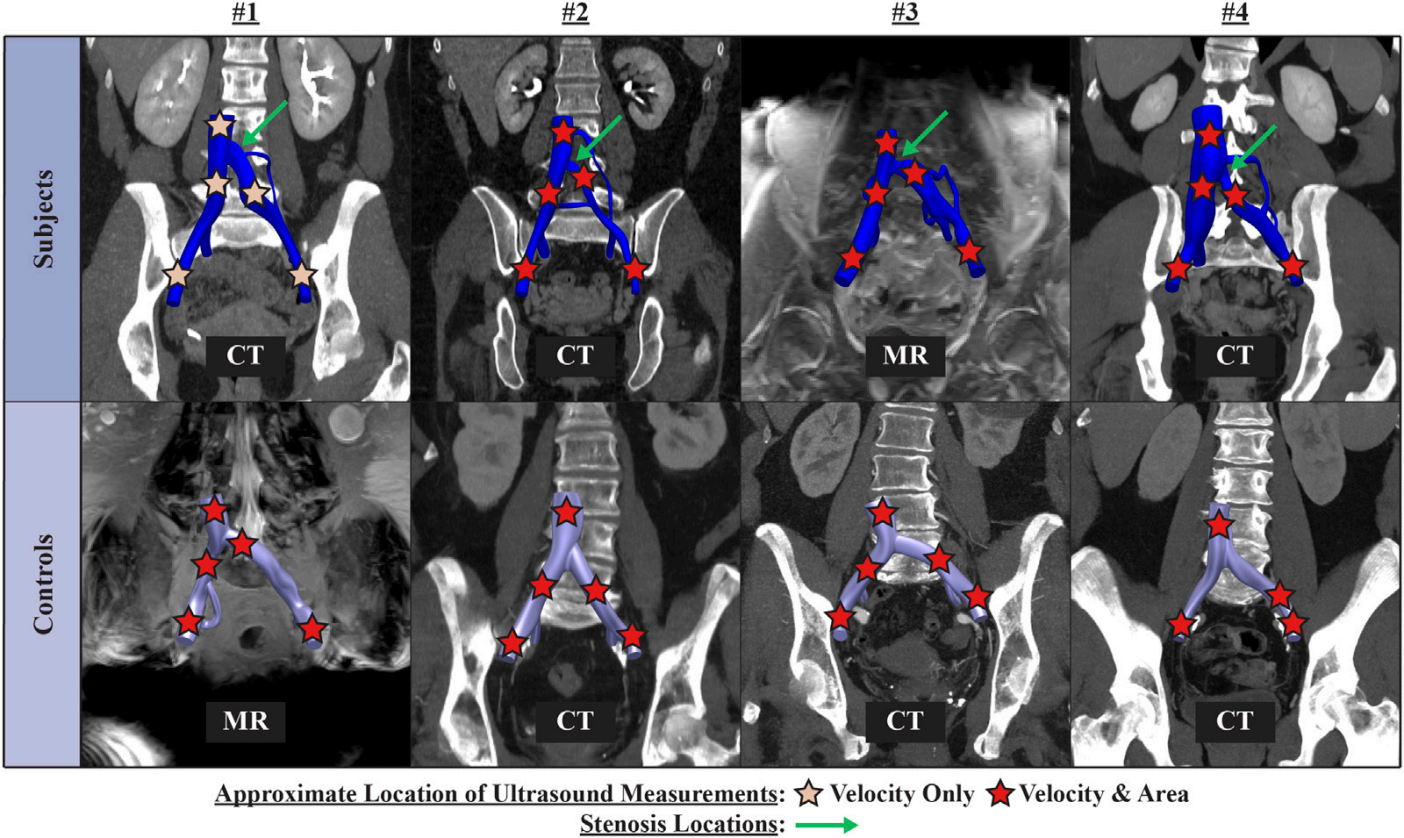
Overview of contrast-enhanced CT or MR image data and approximate location of ultrasound measurements for each patient.

Clinical data on anatomy and flow was comprised of a retrospective, contrast-enhanced CT or MR scan and a prospective ultrasound scan. Figure 1 presents an overview of the CT or MR image data and approximate locations of ultrasound measurements for each patient. All clinical data were acquired in the supine position. Ultrasound acquisition consisted of spectral Doppler waveforms measuring velocity in the sagittal plane and B-mode images measuring cross-sectional area in the transverse plane. Ultrasound measurements were acquired in the infrarenal inferior vena cava (IVC), right common iliac vein (RCIV), LCIV, right external iliac vein (REIV), and left external iliac vein (LEIV).

### 2.2 Computational models of iliac vein hemodynamics

Patient-specific computational models of iliac vein hemodynamics were created using the open-source blood flow modeling software CRIMSON (Arthurs et al., 2021).

#### 2.2.1 Boundary and initial conditions

Given that 1) venous luminal areas are sensitive to a given patient’s hydration status (Meissner et al., 2007) and 2) CT/MR and US scans were not performed on the same day, geometric models of the infrarenal IVC and iliac veins were constructed using a combination of the CT or MR image data and ultrasound area measurements (see **Three-dimensional patient-specific vascular geometries** and **Velocity Validation** in Assi et al., 2023). Furthermore, to minimize the effect of using different imaging techniques for geometric model segmentation on CFD results, all CT and MR scans were evaluated and ensured to be of diagnostic quality by a board-certified radiologist. Vessel walls were prescribed a no-slip (rigid) boundary condition. Inflow waveform boundary conditions were derived using our ultrasound waveform processing protocol (Assi et al., 2023). The respiratory rate was used to set a periodic cycle on the flow waveforms. Inflow waveforms were applied as boundary conditions on the external and internal iliac veins. A 3-element Windkessel lumped-parameter model (RCR) (Xiao et al., 2014) was coupled to the infrarenal IVC and tuned so that the average pressure in the infrarenal IVC was 10 mmHg (Laborda et al., 2014). Blood was modeled as a non-Newtonian fluid (Lynch et al., 2022), with viscosity defined by the Carreau-Yasuda model with parameters μ_∞_ = 0.0035 Pa s, μ_0_ = 0.16 Pa s, n = 0.2128, a = 0.64, and *λ* = 8.2 s (Abraham et al., 2005).

#### 2.2.2 Computational fluid dynamics simulations

Hemodynamic simulations were performed in the Great Lakes high-performance computing cluster at the University of Michigan using 216 cores and a time step size of 0.0001 s for four respiratory cycles. Mesh independence was verified for all simulations. Reported results correspond to finite element meshes of approximately 4 million linear tetrahedral elements. Simulation results were validated by comparing mean ultrasound velocities in the RCIV and LCIV against mean simulated velocities using our validation protocol (Assi et al., 2023). The location of each validation slice was set to the approximate location of the corresponding US measurement.

### 2.3 Data analysis

We performed statistical analyses on clinical and computational metrics comparing the RCIV and LCIV for each group (two-sided, paired Student *t* tests) and comparing the Control and Subject groups for the same vessel (two-sided, homoscedastic Student *t* tests).

#### 2.3.1 Clinical metrics

Mean cross-sectional area and flow were evaluated for RCIV and LCIV. Mean cross-sectional area was calculated as the average cross-sectional area of the vessel wall contours between the common iliac and the internal iliac bifurcations. Mean flow was calculated from the ultrasound waveforms using our ultrasound waveform processing protocol (Assi et al., 2023).

#### 2.3.2 Computational metrics

Shear rate $\left(\dot{\gamma }\right)$ , an index of platelet mechanical activation (Ruggeri, 2007; Sakariassen et al., 2015), was defined as the square root of two times the double contraction of the rate of deformation tensor (Equations 1, 2) (Lynch et al., 2022).

$$D∶=\frac{\left(\nabla u+{\nabla u}^{T}\right)}{2}$$ (1)

$$\dot{\gamma }=\sqrt{2D:D}$$ (2)

Where *D* is the rate of deformation tensor, ∇ is the gradient operation, *u* is the fluid velocity, ^T^ is the transpose operator, $\dot{\gamma }$ is the shear rate, and : is the double contraction operator.

**FIGURE 2:**
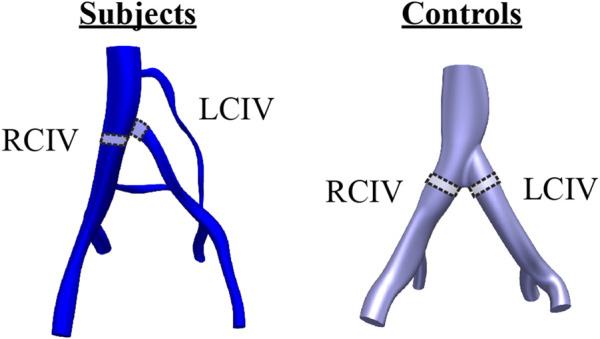
Control volumes in the RCIV and LCIV were used to evaluate computational metrics for each patient. For the Subject group, the LCIV control volume was defined as the volume encompassing the compressed portion of the vessel, where the area is smaller than 90% of the uncompressed proximal and distal luminal areas.

Mean, first quartile (Q1), third quartile (Q3), mean of the peak shear rate over the respiratory cycle (mean peak), and mean LCIV/RCIV shear rate ratio were evaluated. These metrics were extracted from control volumes defined in the RCIV and LCIV of each patient (Figure 2). The rationale for extracting metrics from control volumes is that control volumes provide an average representation of a segment of interest. For the Subject group, the LCIV control volume was defined as the volume encompassing the compressed portion of the vessel, where the area is smaller than 90% of the uncompressed proximal and distal luminal areas. To ensure a fair comparison between LCIV and RCIV, the RCIV control volume was set to start at the same distance from the common iliac bifurcation and to have an identical volume as the corresponding LCIV control volume. For the Control group, the RCIV and LCIV control volumes were set to have the same size and distance from the common iliac bifurcation as the average of the Subject control volumes.

## 3 Results

### 3.1 Patient histories

All four Subjects had a history of left lower extremity DVT. In addition to IVCS, each Subject presented with the following risk factors for DVT. Subject 1 had Factor V Leiden mutation and was taking oral contraceptives. Subject 2 had Factor V Leiden, was a former smoker, had thrombotic events during two different pregnancies, and during COVID-induced pneumonia. Subject 3 had a thrombotic event while immobilized post-surgery. Lastly, Subject 4 had a family history of DVT, was a former smoker, and had recent trauma followed by surgery. DVT dates, locations, risk factors, provoking factors, and treatments for the Subject Group are further outlined in Table 2.

**TABLE 2:** DVT dates, locations, risk factors, provoking factors, and treatments for the Subject Group.

Patient	Date of DVT	Location of DVT	Risk factors	Provoking factor	Treatment
Subject 1	2018	Bilateral PE and L iliofemoral DVT	Factor V Leiden, oral contraceptives	Oral contraceptives	Anticoagulation
Subject 2	2003	L iliofemoral and L tibial DVT	Factor V Leiden, former smoker, pregnancy	Postpartum	Thrombolysis
	2011	R lower leg SVT	Factor V Leiden, former smoker, pregnancy	Postpartum	Unknown
	2021	L iliofemoral and L popliteal DVT	Factor V Leiden, former smoker, COVID	COVID pneumonia	Thrombectomy, IVC filter, LIV stenting, compression stockings, and anticoagulation
Subject 3	2022	L tibial DVT	Recent surgery	Immobilized p/s	Anticoagulation
Subject 4	2022	Bilateral PE, L proximal and mid femoral DVT	Family history of DVT, former smoker, trauma (fall), recent surgery	P/s + trauma	Thrombectomy, compression stockings, and anticoagulation

Patients without a history of IVCS or DVT were selected as controls. These patients had either a recent contrast-enhanced CT or MR in their electronic medical record. The cardiovascular history of each control patient is summarized as follows. Control 1 had an abdominal aortic aneurysm, peripheral artery disease, and hypertension. Control 2 had peripheral artery disease and hypertension. Control 3 had a carotid artery stenosis, gastroduodenal artery aneurysm, and hypertension. Lastly, Control 4 had a carotid artery stenosis, coronary artery disease, peripheral artery disease, a non-ST-elevated myocardial infarction, and hypertension.

### 3.2 Computational boundary conditions

**FIGURE 3:**
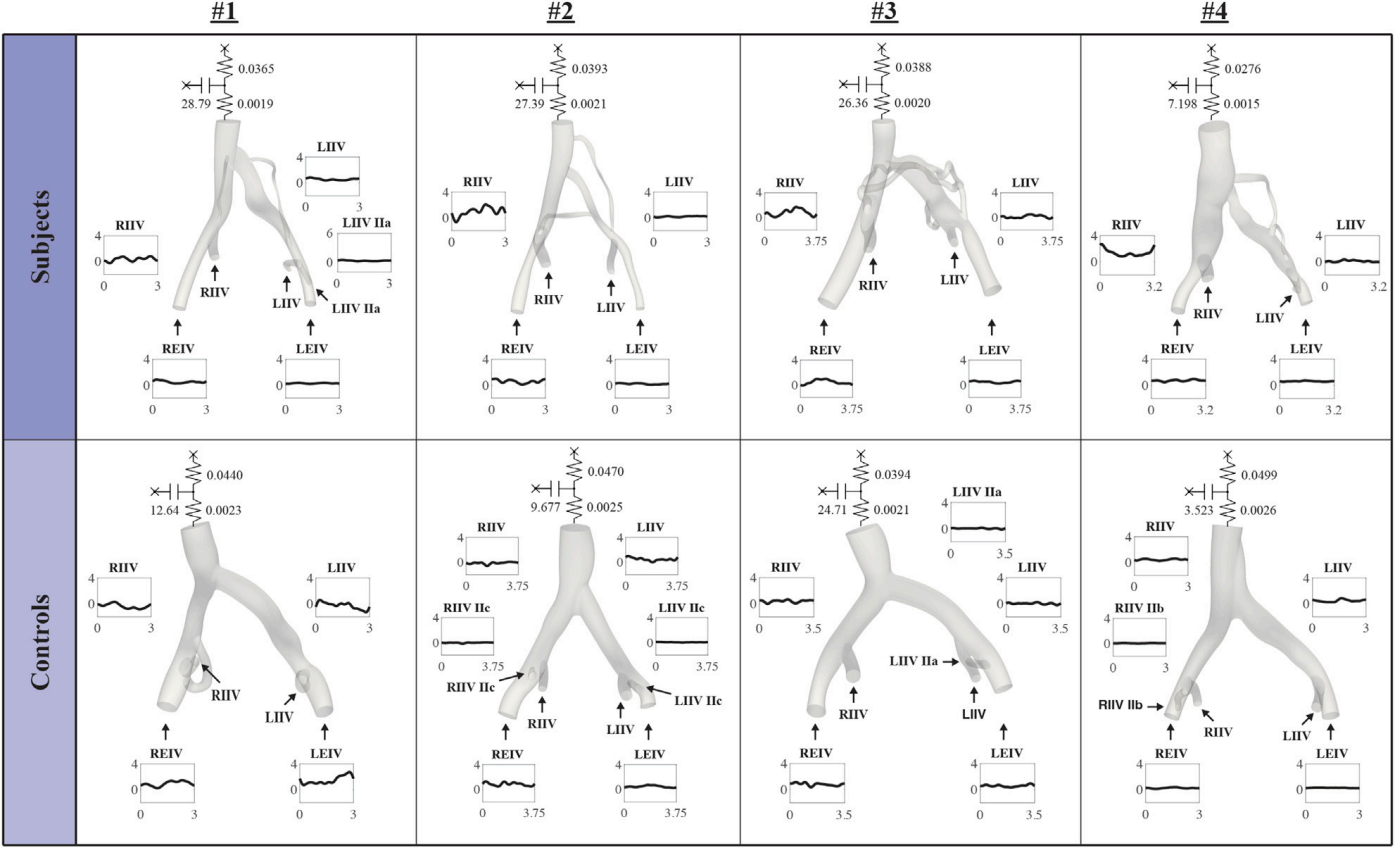
Inflow waveforms and outflow RCR Windkessel boundary conditions.

**FIGURE 4:**
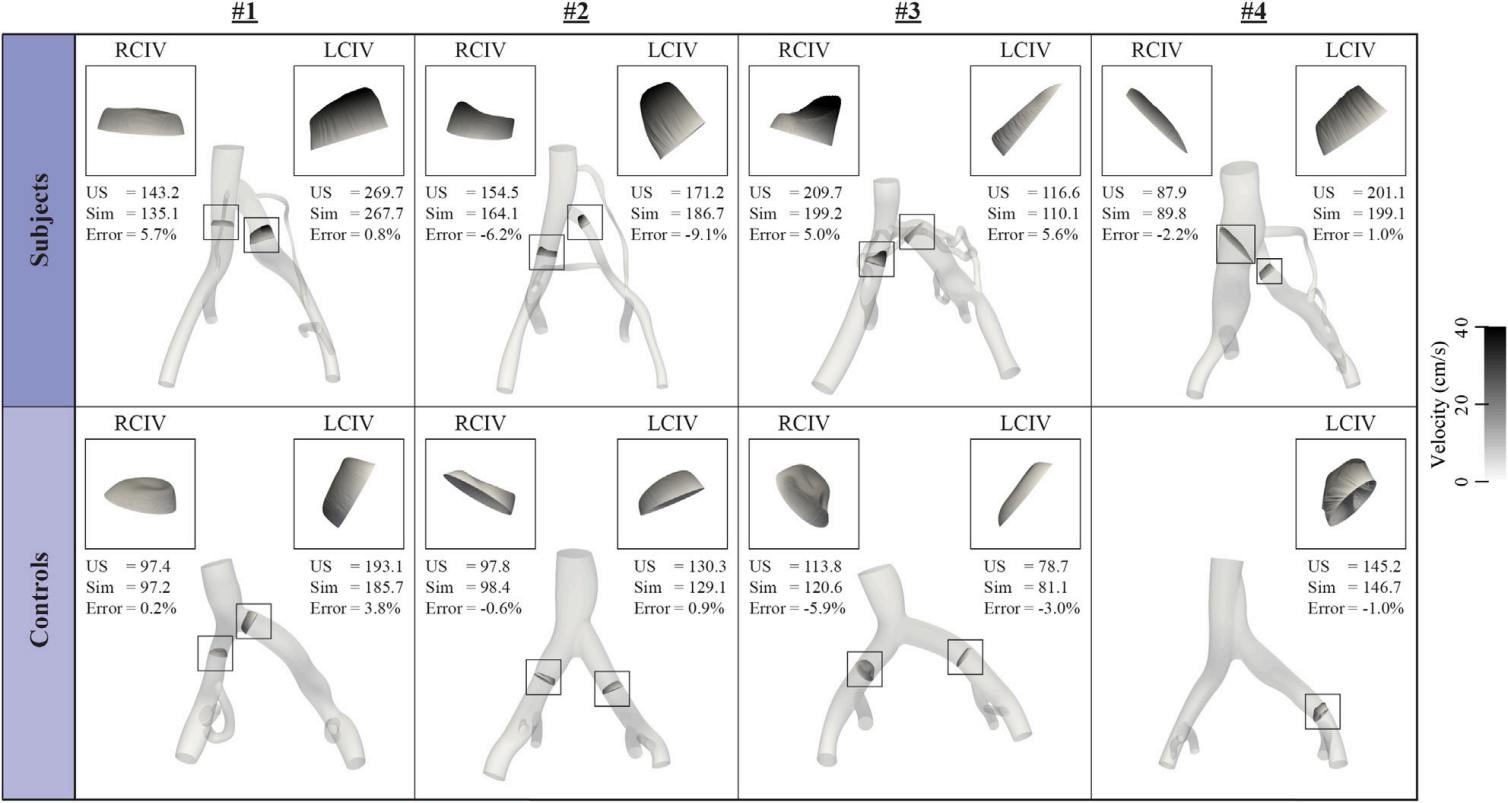
Inflow boundary conditions and vessel areas were iteratively tuned so that simulated velocities in the RCIV and LCIV matched measured ultrasound velocities within 10%.

Figure 3 displays inflow waveforms and outflow RCR Windkessel boundary conditions for the Subject and Control groups. RCR Windkessel parameters, internal iliac flow waveforms, and the area weighting given to define vessel contour areas using CT/MR and US data boundary conditions were iteratively tuned to i) match measured mean flows in the RCIV and LCIV within 2% ii) have an average pressure of approximately 10 mmHg in the IVC outflow and iii) match ultrasound velocities in the RCIV and LCIV within 10% (see **Inflow and outflow boundary conditions**, **Flow calibration**, and **Velocity Validation** in Assi et al., 2023). Figure 4 displays the validation process, comparing measured and simulated mean velocities in the RCIV and LCIV, as well as velocity errors for each patient.

We observed no statistically significant difference between total right leg and total left leg flow for the Subject group (1.46 vs. 0.73 L/min, *p* = 0.141) and as well for the Control group (0.82 vs. 0.91, *p* = 0.718). For the RCR Windkessel parameters (mm-g-s base unites), there was a statistically significant difference between Subject and Control groups proximal resistance (0.00187 vs. 0.00237, *p* = 0.0352) and distal resistance (0.0355 vs. 0.0451, *p* = 0.0352). No statistically significant difference were observed for capacitance (22.4 vs. 12.6, *p* = 0.198) between the two groups.

### 3.3 Analysis of clinical and computational metrics

**FIGURE 5:**
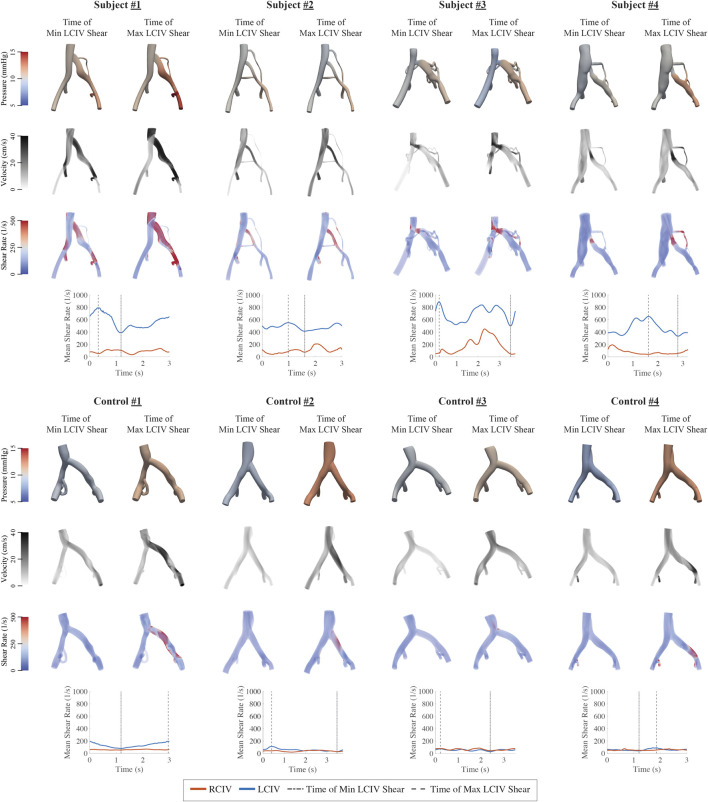
Volume renderings of pressure, velocity, and shear rate in each computational model at the time of minimum and maximum LCIV mean shear rate and plots of RCIV and LCIV mean shear rate during the respiratory cycle.

Clinical and computational metrics are displayed in Table 3. Volume renderings of pressure, velocity, and shear rate in each computational model at the time of minimum and maximum mean LCIV shear rate, as well as plots of mean shear rate during the respiratory cycle are displayed in Figure 5.

**TABLE 3:** Clinical and computational metrics.

	Clinical and computational metrics	Subjects				Average	Std	Controls				Average	Std	*p*-value	
		#1	#2	#3	#4			#1	#2	#3	#4				
LCIV	Mean Cross-Sectional Area (mm^2^)	76.2	30.6	73.4	110.3	72.6	32.7	142.1	131.5	126.0	127.8	131.8	7.2	0.01218	*	
	Mean Flow (L/min)	1.154	0.308	0.469	0.608	0.635	0.367	1.184	1.010	0.694	0.767	0.914	0.225	0.24318	ns	
	Mean Shear Rate (s^−1^)	571	480	688	462	550	103	131	58	53	59	75	37	0.00013	***	
	Shear Rate Q1 (s^−1^)	236	267	348	130	245	90	43	22	18	23	27	11	0.00296	**	
	Shear Rate Q3 (s^−1^)	862	672	986	720	810	142	202	80	81	92	114	59	0.00010	***	
	Mean Peak Shear Rate (s^−1^)	1688	1191	2257	1475	1653	451	503	187	159	175	256	165	0.00114	**	
RCIV	Mean Cross-Sectional Area (mm^2^)	120.4	172.3	113.8	378.7	196.3	124.4	105.6	140.9	165.3	142.3	138.5	24.6	0.39748	ns	
	Mean Flow (L/min)	0.900	1.609	1.352	2.086	1.487	0.495	0.578	0.680	1.238	0.767	0.816	0.292	0.05836	ns	
	Mean Shear Rate (s^−1^)	89	103	183	78	113	48	59	39	64	55	54	11	0.05209	ns	
	Shear Rate Q1 (s^−1^)	35	41	51	28	39	10	22	18	26	33	25	6	0.05312	ns	
	Shear Rate Q3 (s^−1^)	132	146	249	85	153	69	69	58	92	69	72	14	0.06149	ns	
	Mean Peak Shear Rate (s^−1^)	254	385	923	616	545	293	316	100	260	174	213	95	0.07478	ns	
	Mean LCIV/RCIV Shear Rate Ratio	7.21	5.59	6.02	7.43	6.56	0.90	2.21	1.58	0.84	1.09	1.43	0.60	0.00008	****	

#### 3.3.1 RCIV vs. LCIV metrics

**FIGURE 6:**
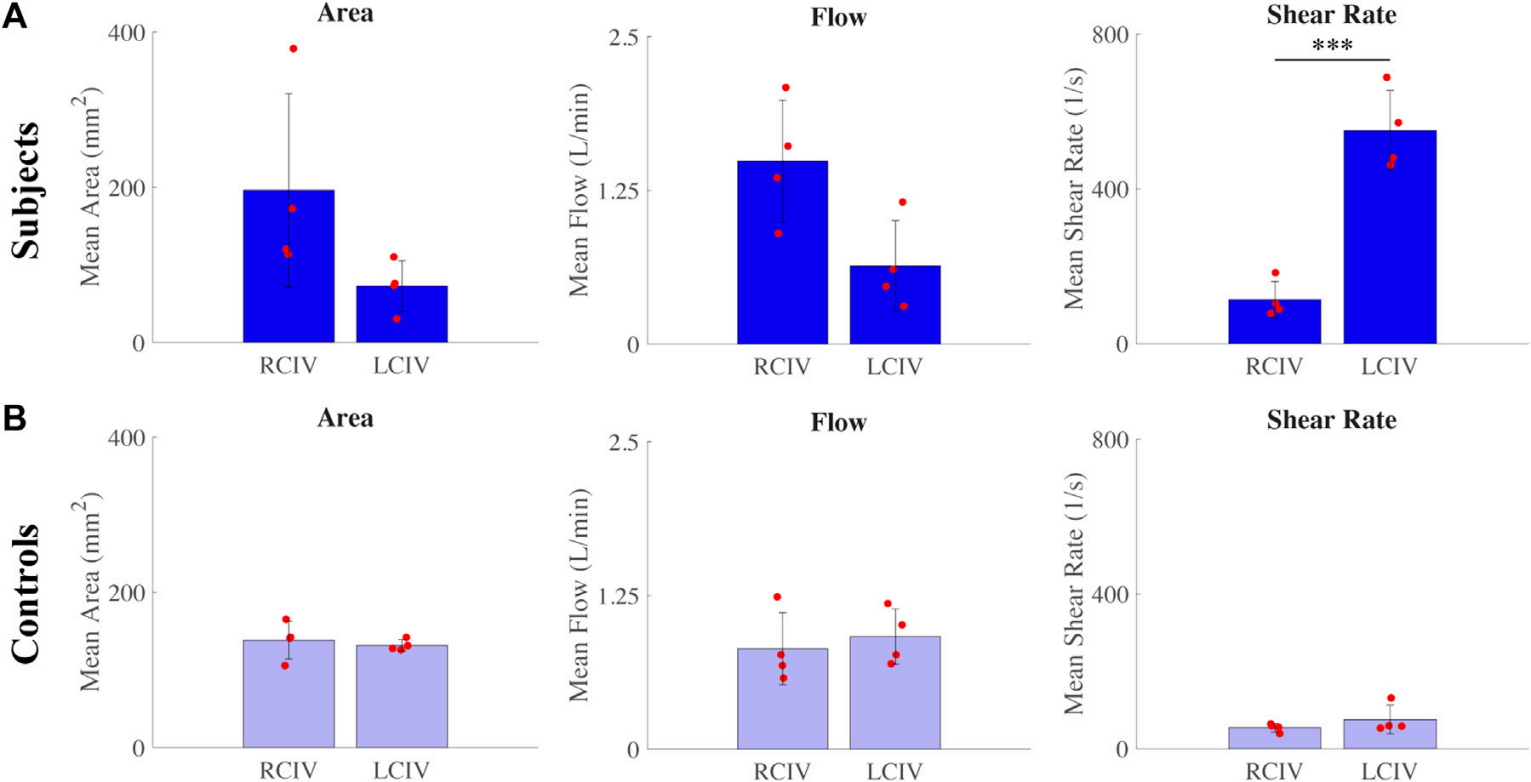
Differences between RCIV and LCIV mean area, flow, and shear rate for the **(A)** Subject group and **(B)** Control group.

For the Subject group, differences between RCIV and LCIV mean area (mm^2^), flow (L/min), and shear rate (s^−1^) are displayed in Figure 6A. No statistically significant differences were observed for mean area (RCIV = 196.3 and LCIV = 72.6, *p* = 0.104) and mean flow (RCIV = 1.5 and LCIV = 0.6, *p* = 0.117). A significant difference was observed for mean shear rate (RCIV = 113 and LCIV = 550, *p* = 0.0009).

For the Control group, differences between RCIV and LCIV mean area (mm^2^), flow (L/min), and shear rate (s^−1^) are displayed in Figure 6B. No statistically significant differences were observed for mean area (RCIV = 138.5 and LCIV = 131.8, *p* = 0.700), mean flow (RCIV = 0.8 and LCIV = 0.9, *p* = 0.718), and mean shear rate (RCIV = 54 and LCIV = 75, *p* = 0.329).

#### 3.3.2 Comparison of metrics between Control and Subject groups

**FIGURE 7:**
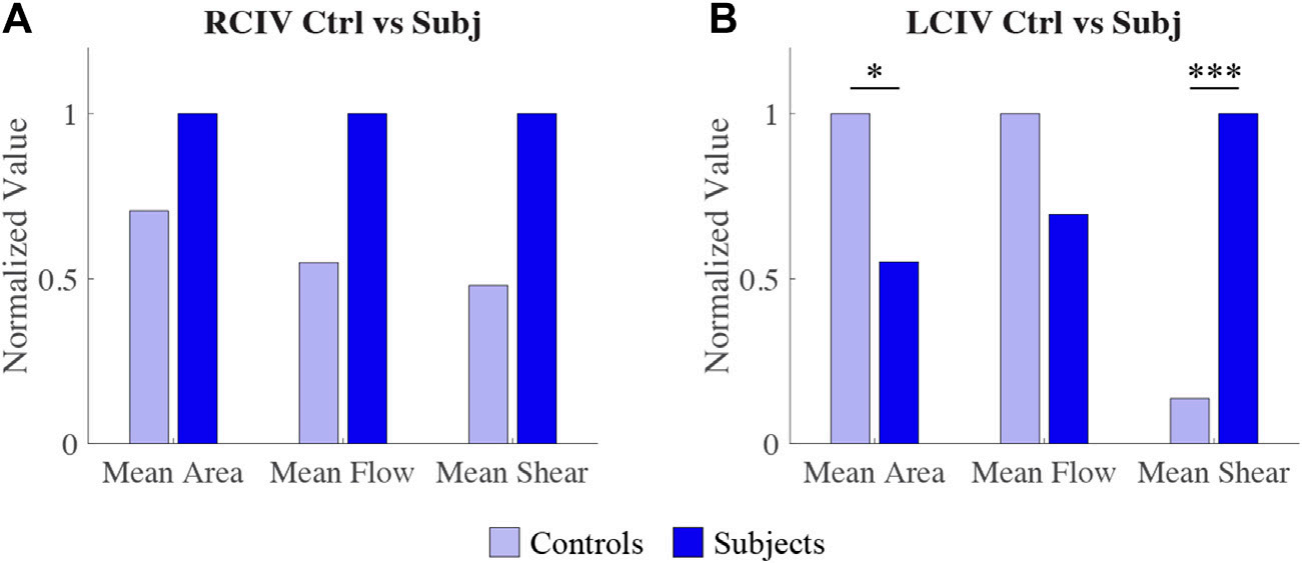
Comparison of normalized differences in RCIV metrics **(A)** and LCIV metrics **(B)** between Control and Subject groups.

For the RCIV, normalized differences in mean area, flow, and shear rate between the Control and Subject groups are displayed in Figure 7A. No statistically significant differences were observed for area (*p* = 0.397), flow (*p* = 0.0584), or shear rate (*p* = 0.0521).

For the LCIV, normalized differences in mean area, flow, and shear rate between the Control and Subject groups are displayed in Figure 7B. Statistically significant differences were observed for area (*p* = 0.0122) and shear rate (*p* = 0.0001). No statistically significant differences were observed for flow (*p* = 0.243).

#### 3.3.3 LCIV/RCIV shear rate ratio

**FIGURE 8:**
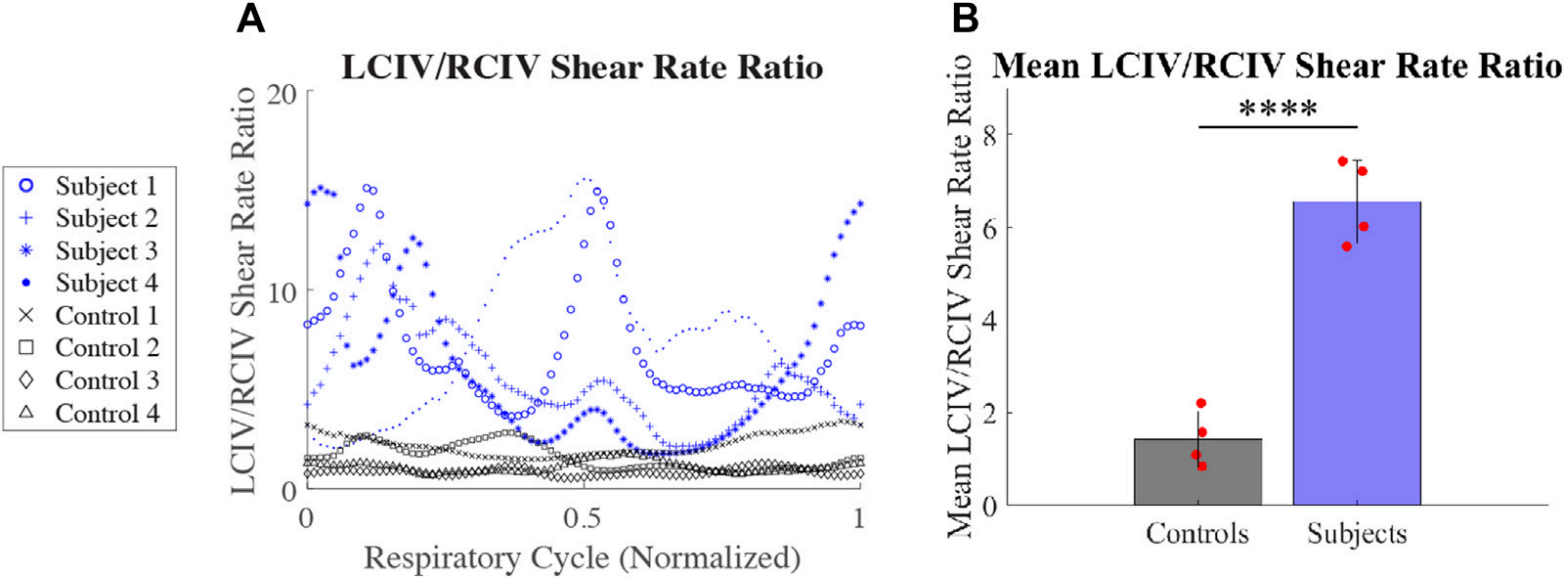
**(A)** LCIV/RCIV shear rate ratio over time during normalized respiratory cycle for each patient. **(B)** Mean LCIV/RCIV shear rate ratios for the Control and Subject groups.

LCIV/RCIV shear rate ratio over time during normalized respiratory cycle is displayed in Figure 8A for each patient. Mean LCIV/RCIV shear rate ratios for the Control and Subject groups are displayed in Figure 8B. Mean LCIV/RCIV shear rate ratio for the Control group was 1.43 ± 0.6 and 6.56 ± for the Subject group (*p* = 0.00008).

## 4 Discussion

In this work we have built and validated high-resolution hemodynamic models for a cohort of 4 IVCS and four healthy patients (Figure 4). Our goal was to establish hemodynamic patterns differentiating stenosed and healthy veins and to identify metrics that could be used for risk-stratification of IVCS patients and for providing a baseline for follow-up clinical assessment of treated patients.

From our computational models, we observed that there is a large variability in flow and cross-sectional area within each group. Therefore, it is not clear whether flow or area can provide the diagnostic sensitivity to identify statistically significant differences in iliac vein hemodynamics between Subjects and Controls. This is illustrated by the fact that despite right leg inflow being twice that of the left leg inflow in the Subject group, this difference was not statistically significant (*p* = 0.141). Figures 6, 7 also show that despite seemingly large differences in flow and area, the differences are not statistically significant between Subjects and Controls, or between RCIV and LCIV. The lack of statistical significance of these results, however, may be due to the low number of samples.

Because shear rate depends on both flow and area, our results suggest that shear rate could serve as a more robust metric to stratify Subjects *versus* Controls. In the Subject group, the mean shear rate in the LCIV was significantly higher than in the RCIV (*p* = 0.0009). In contrast, no statistically significant differences in mean shear rate were observed between LCIV and RCIV (*p* = 0.329) in the Control group. Furthermore, when comparing mean shear rates in the LCIV between the Subject and Control groups, significantly higher values were observed in the Subject group (550 vs. 75 s^−1^, *p* = 0.0001, Table 3). Despite the statistical significance of these results, it is likely that the determination of shear rate is affected by more sources of uncertainty (namely, geometry segmentation and CFD assumptions) than routine US area and flow measurements.

IVCS hemodynamics may be affected by variations in hydration status, muscle tone, cardiac output, and degree and anatomy of vessel collateralization. Thus, shear rates measured at one time point will likely differ from values measured several hours or weeks later. In view of this temporal variability in shear rate, in this paper we proposed the LCIV/RCIV shear rate ratio as a standardized and highly interpretable metric that uses the contralateral vessel as a control. For example, the Control group presented with a mean LCIV/RCIV shear rate ratio of 1.43. Conversely, the Subject group presented with a mean LCIV/RCIV shear rate ratio of 6.56. Furthermore, the LCIV/RCIV shear rate ratio rendered the most statistically significant difference between Subjects and Controls of all reported metrics in our study (*p* = 0.00008).

Beyond providing a metric that incorporates information on both flow and area through a vessel, shear rate has additional implications with regards to thrombosis. In the arterial system, elevated shear rates are known to contribute to thrombus initiation by increasing platelet-platelet adhesion (Ruggeri, 2007; Sakariassen et al., 2015). *In-vitro* studies have further demonstrated that shear-dependent thrombosis initiation is triggered at shear rates of around 1,000 s^−1^ (Ruggeri, 2007). Given that the venous system is assumed to be a low flow, low shear rate system, shear rate activation of platelets has typically been overlooked as a potential factor contributing to thrombosis initiation in IVCS patients. We observed, however, that every Subject presented with mean peak shear rates well over the 1,000 s^−1^ threshold (average values of 1,653 s^−1^), whereas every Control presented with much smaller mean peak shear rates, well below that threshold (average values of 256 s^−1^) (Table 3). These results suggest that thrombus initiation in IVCS patients may be affected by shear rates that more closely resemble those found in arterial thrombosis.

These findings are especially relevant given the recent increase in venous stenting (Keegan et al., 2023) after the addition of new venous-specific stents to the market. Non-thrombotic iliac vein stenting is now commonly performed, but it is unclear in which patients stenting may provide a long-term benefit in thrombosis risk reduction by alleviating shear rate or other parameters that may influence thrombus formation. Investigation of shear rate in IVCS patients before thrombotic events occur may help identify a population in whom prophylactic stenting is warranted to prevent future thrombotic events and limit overuse of such invasive procedures.

Furthermore, an increased incidence of DVT in IVCS patients has been reported (Raju and Neglen, 2006; Thijs et al., 2010), suggesting a potential role of IVCS in thrombus initiation. It is generally acknowledged that IVCS plays a permissive role in DVT. That is, patients with IVCS will remain relatively asymptomatic until an additional “insult” appears (Raju and Neglen, 2006). This permissive and multi-causal nature of venous thrombosis is consistent with Virchow’s triad, which states that a combination of two or more “insults” among elevated or reduced blood flow, endothelial injury, and hypercoagulability are needed for thrombus initiation (Virchow, 1859). In this study, in addition to the observed elevated shear rates, all Subjects presented with an acute insult such as oral contraceptives, pregnancy, COVID, immobilization post-surgery, and trauma.

**FIGURE 9:**
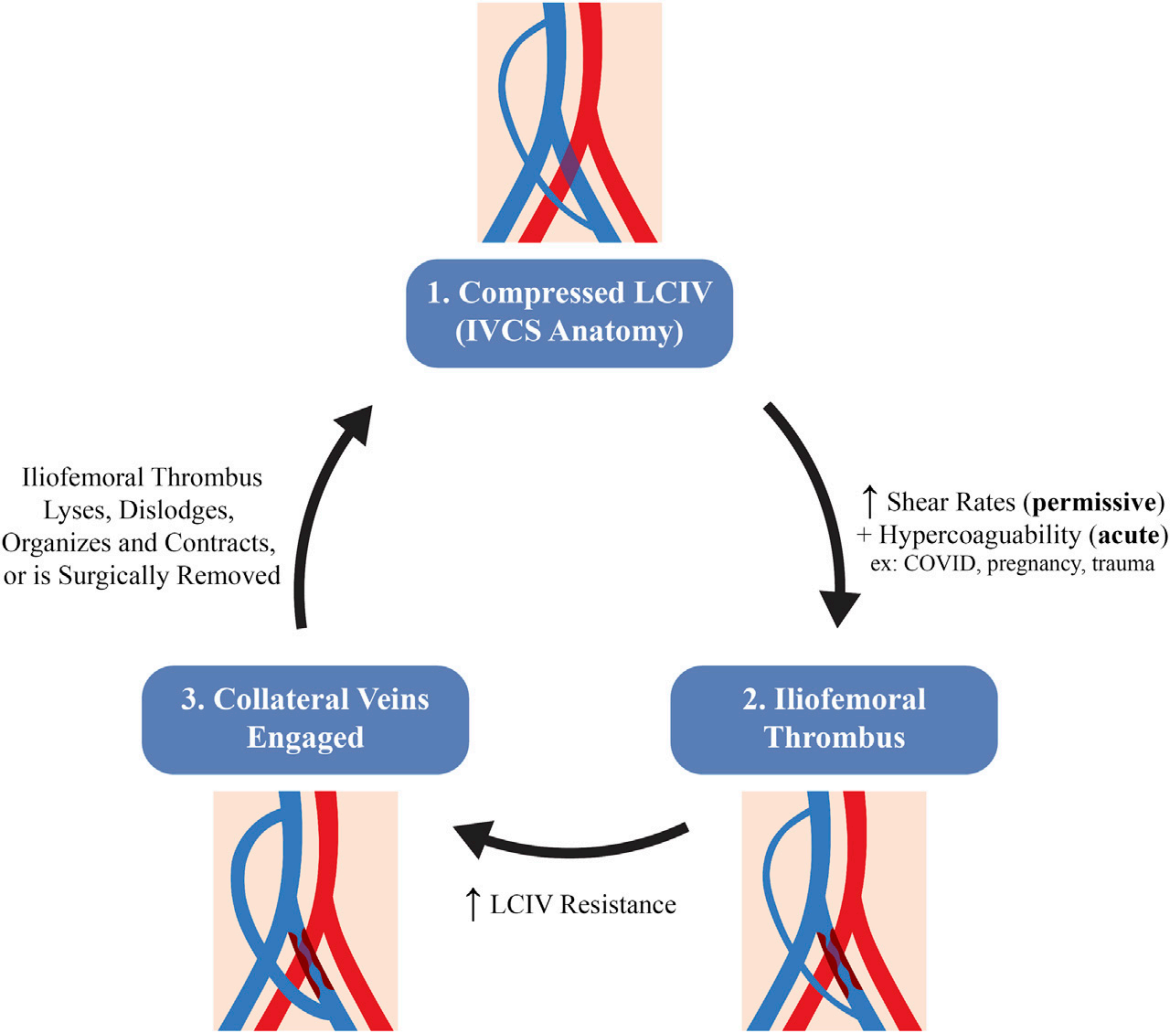
Hypothesized schematic of the stages in venous hemodynamics of an IVCS patient during the thrombus formation-resolution cycle.

IVCS patients who develop DVT typically undergo cycles of thrombus formation and resolution (Meissner et al., 1995). Therefore, the timing of each patient in the thrombus formation-resolution cycle will impact their observed iliac vein hemodynamics, as detailed in Figure 9. For example, a patient with a significant LCIV compression will first have increased shear rates which serve as a permissive thrombotic pathology. With an additional insult, a thrombus will form in the iliofemoral region. Second, the iliofemoral thrombus will increase LCIV resistance which then diverts LCIV flow to trans-sacral, lumbar, or paravertebral collaterals, decreasing shear rates in the LCIV. Third, the thrombus may be cleared by pharmacological or pharmacomechanical thrombolysis, mechanical or surgical thrombectomy, or the thrombus may undergo spontaneous resolution, returning iliac vein hemodynamics back to their initial state. Given the hemodynamic changes during the formation and resolution of iliac thrombi, we submit that the severity of symptoms which IVCS patients present with is highly dependent on their timing in the DVT formation-resolution cycle, as well as the original degree of LCIV compression and the extent to which collateral pathways are able to shunt left leg flow.

Lastly, the methodology outlined in this work demonstrates a workflow to identify functional metrics for risk-stratification of IVCS patients using validated CFD simulations. This methodology can be applied to other vascular pathologies in which disease severity or the decision whether or not to operate is difficult to ascertain using traditional diagnostic techniques. Some relevant examples of such pathologies are peripheral artery disease or carotid artery disease for which we believe that CFD-based approaches can be similarly leveraged to inform pre-operative risk stratification of patients.

## 5 Conclusion

In this study, we built and validated high resolution computational fluid dynamics models of the iliac veins for 4 patients with IVCS and 4 patients with healthy veins. Our analyses revealed that IVCS patients experience shear rates more typical of the arterial system; IVCS patients presented with a mean LCIV/RCIV shear rate ratio 4.6 times higher than that of healthy patients. We propose that the mean LCIV/RCIV shear rate ratio may be a suitable metric for risk stratification of IVCS patients with moderate, yet symptomatic compression in which clinical treatment is highly variable. More investigation is needed to assess the prognostic value of shear rate as a clinical metric and to understand the mechanisms of thrombus formation in IVCS patients.

### 5.1 Limitations

A small cohort of patients were included in this study (four Subjects, four Controls), which casts uncertainty onto the statistical significance of the reported relationships. Furthermore, this study was single center and non-randomized. All patients were Caucasian and mostly female, which is not representative of the average population, although females are more likely to present with IVCS-related complications than males (Kaltenmeier et al., 2018). Furthermore, the Control group was older than the Subject group. No other statistically significant differences in patient demographics were observed between the two groups.

Since patients in the Subject group had already presented with acute or chronic thrombotic events, their hemodynamics may differ from patients with uncomplicated IVCS due to the changes in stenosis length and vessel wall stiffness that arise from post-thrombotic inflammatory responses. Furthermore, since Control patients were required to have a recent CT or MR and an upcoming ultrasound scan, all patients in the Control group presented with arterial disease. However, none of the Controls presented with any venous disease and thus we considered their venous hemodynamics to be a good surrogate of those in healthy patients. However, we submit that better characterization of venous hemodynamics of healthy patients (no cardiovascular disease whatsoever) is further needed to confirm generalizability of our results.

Lastly, the limitations outlined in our iliac vein computational modeling protocol (Assi et al., 2023) apply to these analyses, including the rigid wall assumption used to model the iliac veins, the uncertainty in area measurements, and the underestimation of the LCIV/RCIV shear rate ratio in the Subject group. One computational modeling limitation not discussed in Assi et al., 2023 is that the use of different imaging techniques (CT and MR) for the segmentation could potentially affect comparisons of CFD results. Furthermore, because no US velocity or area measurements were acquired in the RCIV of Control 4, RIIV boundary conditions were not tuned for Control 4, a limitation that could also potentially affect comparisons of CFD results.

## Data Availability

The datasets presented in this article are not readily available because the original data has personally identifiable information. All computational data will be made available upon request. Requests to access the datasets should be directed to ismael@umich.edu.

## References

[B1] Abraham F. Behr M. Heinkenschloss M. (2005). Shape optimization in steady blood flow: a numerical study of non-Newtonian effects. Comput. Methods Biomechanics Biomed. Eng. 8 (2), 127–137. 10.1080/10255840500180799 16154876

[B2] Arthurs C. J. Khlebnikov R. Melville A. Marčan M. Gomez A. Dillon-Murphy D. (2021). CRIMSON: an open-source software framework for cardiovascular integrated modelling and simulation. PLoS Comput. Biol. 17 (5), e1008881. 10.1371/journal.pcbi.1008881 33970900 PMC8148362

[B3] Assi I. Z. Lynch S. R. Samulak K. Williams D. M. Wakefield T. W. Obi A. T. (2023). An ultrasound imaging and computational fluid dynamics protocol to assess hemodynamics in iliac vein compression syndrome. J. Vasc. Surg. Venous Lymphatic Disord. 11 (5), 1023–1033.e5. 10.1016/j.jvsv.2023.05.017 37353157

[B4] Cheng L. Zhao H. Zhang F.-X. (2017). Iliac vein compression syndrome in an asymptomatic patient population: a prospective study. Chin. Med. J. 130 (11), 1269–1275. 10.4103/0366-6999.206341 28524824 PMC5455034

[B5] Engelhorn A. L. D. V. Lima L. de B. Werka M. J. S. Engelhorn A. V. V. Bombardelli D. A. R. da Silva L. D. O. (2021). Left common iliac vein compression identified by vascular ultrasonography in asymptomatic women: does standing position influence diagnosis? J. Vasc. Bras. 20, e20200188. 10.1590/1677-5449.200188 34267789 PMC8256875

[B6] Hng J. Z. K. Su S. Atkinson N. (2021). May–Thurner syndrome, a diagnosis to consider in young males with no risk factors: a case report and review of the literature. J. Med. Case Rep. 15 (1), 141–147. 10.1186/s13256-021-02730-8 33736685 PMC7977182

[B7] Kaltenmeier C. T. Erben Y. Indes J. Lee A. Dardik A. Sarac T. (2018). Systematic review of May-Thurner syndrome with emphasis on gender differences. J. Vasc. Surg. Venous Lymphatic Disord. 6 (3), 399–407.e4. 10.1016/j.jvsv.2017.11.006 29290600

[B8] Keegan A. Bose S. Dun C. McDermott K. Stonko D. O’Banion L. A. (2023). Temporal trends in venous stenting practice patterns in a US commercial database. J. Vasc. Surg. 78 (4), e117–e118. 10.1016/j.jvs.2023.08.056

[B9] Kibbe M. R. Ujiki M. Goodwin A. L. Eskandari M. Yao J. Matsumura J. (2004). Iliac vein compression in an asymptomatic patient population. J. Vasc. Surg. 39 (5), 937–943. 10.1016/j.jvs.2003.12.032 15111841

[B10] Laborda A. Sierre S. Malvè M. De Blas I. Ioakeim I. Kuo W. T. (2014). Influence of breathing movements and Valsalva maneuver on vena caval dynamics. World J. radiology 6 (10), 833–839. 10.4329/wjr.v6.i10.833 PMC4209428 25349666

[B11] Labropoulos N. Borge M. Pierce K. Pappas P. J. (2007). Criteria for defining significant central vein stenosis with duplex ultrasound. J. Vasc. Surg. 46 (1), 101–107. 10.1016/j.jvs.2007.02.062 17540535

[B12] Li C. Zhan Y. Wang Z. Gao Y. Ye K. Lu X. (2023). Effect of stent treatment on hemodynamics in iliac vein compression syndrome with collateral vein. Med. Eng. Phys. 115, 103983. 10.1016/j.medengphy.2023.103983 37120173

[B13] Lynch S. Nama N. Figueroa C. A. (2022). Effects of non-Newtonian viscosity on arterial and venous flow and transport. Sci. Rep. 12 (1), 20568. 10.1038/s41598-022-19867-1 36446813 PMC9709089

[B14] May R. Thurner J. (1957). The cause of the predominantly sinistral occurrence of thrombosis of the pelvic veins. Angiology 8 (5), 419–427. 10.1177/000331975700800505 13478912

[B15] Meissner M. H. Caps M. T. Bergelin R. O. Manzo R. A. Strandness D. E. J. (1995). Propagation, rethrombosis and new thrombus formation after acute deep venous thrombosis. J. Vasc. Surg. 22 (5), 558–567. 10.1016/s0741-5214(95)70038-2 7494356

[B16] Meissner M. H. Moneta G. Burnand K. Gloviczki P. Lohr J. M. Lurie F. (2007). The hemodynamics and diagnosis of venous disease. J. Vasc. Surg. 46 (6 Suppl. L), 4–24. 10.1016/j.jvs.2007.09.043 18068561

[B17] Oğuzkurt L. Ozkan U. Tercan F. Koç Z. (2007). Ultrasonographic diagnosis of iliac vein compression (May-Thurner) syndrome. Diagnostic interventional radiology (Ankara, Turk. 13 (3), 152–155. 17846991

[B18] Raju S. Neglen P. (2006). High prevalence of nonthrombotic iliac vein lesions in chronic venous disease: a permissive role in pathogenicity. J. Vasc. Surg. 44 (1), 136–144. 10.1016/j.jvs.2006.02.065 16828437

[B19] Ruggeri Z. M. (2007). The role of von Willebrand factor in thrombus formation. Thrombosis Res. 120 (Suppl. 1), S5–S9. 10.1016/j.thromres.2007.03.011 PMC2702526 17493665

[B20] Sakariassen K. S. Orning L. Turitto V. T. (2015). The impact of blood shear rate on arterial thrombus formation. Future Sci. OA 1 (4), FSO30. 10.4155/fso.15.28 28031903 PMC5137878

[B21] Thijs W. Rabe K. F. Rosendaal F. R. Middeldorp S. (2010). Predominance of left-sided deep vein thrombosis and body weight. J. Thrombosis Haemostasis 8 (9), 2083–2084. 10.1111/j.1538-7836.2010.03967.x 20586917

[B22] Virchow R. (1859). Rudolf Virchow: archiv für pathologische Anatomie und Physiologie und für klinische Medicin. Berlin, Germany: De Gruyter. PMC5182399 30163712

[B23] Wang H. Jia W. Xi Y. Li Y. Fan Y. Deng X. (2022). Morphometric and hemodynamic analysis of the compressed iliac vein. J. endovascular Ther. official J. Int. Soc. Endovascular Specialists, 15266028221134895. 10.1177/15266028221134895 36408873

[B24] Xiao N. Alastruey J. Figueroa C. A. (2014). A systematic comparison between 1-D and 3-D hemodynamics in compliant arterial models. Int. J. Numer. methods Biomed. Eng. 30 (2), 204–231. 10.1002/CNM.2598 PMC4337249 24115509

